# Chinese Herbal Formula Feilin Vaginal Gel Prevents the Cervicitis in Mouse Model

**DOI:** 10.1155/2019/4168126

**Published:** 2019-01-10

**Authors:** Xin Mao, Ronghua Zhao, Rongmei Yao, Shanshan Guo, Lei Bao, Yingjie Gao, Jing Sun, Yanyan Bao, Yujing Shi, Xiaolan Cui

**Affiliations:** ^1^Institute of Chinese Materia Medica, China Academy of Chinese Medical Sciences, Beijing 100700, China; ^2^College of Traditional Chinese Medicine, North China University of Sciences and Technology, Hebei 063210, China

## Abstract

Cervicitis is a common sexually transmitted disease. In recent years, the abuse of antibiotic in the treatment of cervicitis results in the emergence of antibiotic-resistant bacteria; alternative strategies are needed to be developed. In this research, we investigated the effects of Feilin Vaginal Gel (FVG), a Chinese herbal formula, on the treatment of cervicitis. Two cervicitis models were optimized using BALB/c mouse; one* in vitro* model was established in HeLa cells. In* Chlamydia trachomatis*-induced cervicitis model, the high level of bacterial loads, the inflammation in tissue, and the cytokines in serum could be observed. With the administration of FVG, the bacterial loads in cervical mucus and cervix tissue could be significantly inhibited in dose-dependent manners. The pathological injury of cervix and vagina, as well as the levels of IL-2, IL-17, and MCP-1 in serum, could be mitigated by FVG. FVG reduced the number of inclusion induced by* C. trachomatis* in HeLa cells. In addition, the histological damage in* Escherichia coli* and* Staphylococcus aureus*-induced cervicitis model could be reduced by FVG. These results suggest that FVG is capable of treating cervicitis through the inhibition of pathogens and the regulation of host immune responses. FVG may contribute as an alternative agent for the treatment of cervicitis.

## 1. Introduction

Cervicitis is a common sexually transmitted disease with an inflammatory condition of the uterine cervix [1]. The infection of cervix starts from lower genital tract (vagina) and then develops into the pelvic inflammatory disease with ascending infection of the upper genital tract (uterus and fallopian tubes) and peritoneal cavity [2]. Cervicitis occurred frequently as an asymptomatic infection. Abnormal cervical or vaginal mucopurulent discharge and cervical ectopy may be the signs and symptoms of cervicitis in some patients [3]. However, serious cervicitis can lead to further infertility and ectopic pregnancy. Cervicitis is considered to be associated with the transmission of HIV infection and the development of cervical carcinomas [4].

Cervicitis can be induced by various pathogens such as* Chlamydia trachomatis*,* Neisseria gonorrhea*,* Mycoplasma genitalium*,* Mycoplasma hominis*,* Ureaplasma urealyticum*,* Trichomonas*, Herpes simplex virus, cytomegalovirus, and adenovirus [4].* C. trachomatis* infection is reported to be the most frequent cause of cervicitis. More than 10% of women with cervicitis are diagnosed to be infected by* C. trachomatis* and the number of these infections continues to increase over the past decades [5].* C. trachomatis* is Gram-negative obligate intracellular bacterium. Two forms of* C. trachomatis* can be found in its developmental cycle. Attachment of elementary bodies (EBs) to host cells mediates the invasion of* C. trachomatis*. Inside the host cells,* C. trachomatis* forms the reticulate bodies (RBs) and then RBs replicate within the cytoplasmic vacuole and finally form the inclusion [6].

Chlamydia can persist for a long time in uterine cervix without symptom. The infection may resolve spontaneously without treatment or may cause cervicitis. The clearance of* C. trachomatis* requires the responses of Th1 immunity [7, 8]. However, the responses of adaptive immunity may have a double-edged nature and bring tissue damage. The management of* C. trachomatis* infection is based on the treatment of the patients and their sexual partners with macrolides. The abuse of antibiotic and false-positive diagnose may contribute to the emergence of antibiotic-resistant organisms. The antibiotic-resistant bacteria, on the other hand, lead to treatment failure [4, 5, 9]. After treatment with the antibiotic, 10%-20% of patients will suffer from reinfection within one year, which may be due to the absence of protective immunity against Chlamydia [10]. Considering these conditions, alternative strategies are needed to be developed.

Traditional Chinese Medicine (TCM) has a long history in treating gynecological diseases [11]. Feilin Vaginal Gel (FVG) is developed from a clinically used Chinese medicine formula that is used to treat the mucopurulent cervicitis. FVG consists of the following herbal medicines: Gentianae radix et rhizoma, Stemonae radix, Fraxini cortex, Paeoniae radix rubra, Dictamni cortex, and Glycyrrhizae radix et rhizome. According to the theory of TCM: FVG can be used to treat the accumulation of dampness and toxic materials induced abnormal vaginal discharge, such as profuse, fetid and yellowish leukorrhea, itching, and pain around the external genitalia [12]. The extraction, preparation, and quality control of FVG had been studied. The concentrations of gentiopicroside and paeoniflorin in FVG were analyzed to be approximately 5.90 mg/g and 8.96 mg/g, respectively [12].

In the present study, we tested the hypothesis that FVG could treat the cervicitis effectively. Mouse cervicitis models were established to evaluate the treatment of FVG. In the* C. trachomatis* infected mouse model, the bacterial loads, the pathological injury, and the levels of MCP-1, IL-2, and IL-17 in serum were evaluated. The inclusions in HeLa cells induced by* C. trachomatis* were enumerated* in vitro*. In addition, after being infected by the mixture of* Escherichia coli* and* Staphylococcus aureus*, the treatment of FVG was evaluated through the histological examination of the cervix.

## 2. Materials and Methods

### 2.1. Preparation of FVG

The six herbal medicines were extracted three times with boiling water and precipitated with alcohol to 70% (v/v) ethanol. The supernatant was concentrated with the rotary evaporator to a relative density of 1.30-1.35 (50°C) to get the Feilin extraction (FE, 5.62 g/g). The gel base was the mixture of carbopol 941 and xanthan gum. The extraction was mixed with the gel base to get FVG. The drug loading of FVG was 2.5 g/g, 1.25 g/g, and 0.625 g/g.

### 2.2. Bacterial Strains and Host Cell

*Chlamydia trachomatis* mouse pneumonitis strain Nigg II (ATCC® VR-123™),* Escherichia coli* (ATCC® 25922™), and* Staphylococcus aureus* (ATCC® 25923™) were obtained from American Type Culture Collection.* E. coli* and* S. aureus* were cultured in nutrient broth. Human cervix epithelial cell line HeLa (ATCC® CCL-2™) was purchased from Culture Collection of Chinese Academy of Medical Sciences and cultured in RPMI 1640 medium containing 10% FBS at 37°C in the presence of 5% CO_2_.* C. trachomatis* N was propagated in HeLa cells. Before infection, infected HeLa cells (10^5^ cells/mL) were disrupted and centrifuged. The supernatant was collected to get purified elementary bodies (EBs), which can be used to infect mice and HeLa cells directly.

### 2.3. Animal Infections and Treatment

120 female BALB/c mice (18-20 g) were purchased from Charles River Laboratories China (Beijing, China). Animals were housed in a BSL2 barrier animal facility. All animal experimental procedures described here were by the permission of Institute of China Academy of Chinese Medical Sciences, Chinese Materia Medica, Ethic Committee. The ethic approval reference number is 20162019.

**Figure 1:**
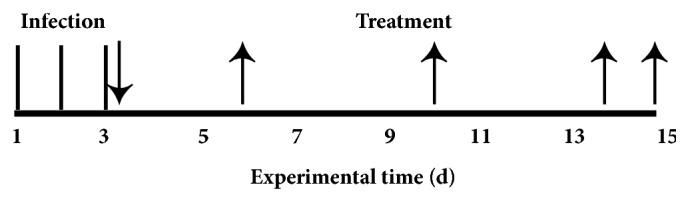
Schematic of the experimental timeline used to establish the cervicitis models.

The cervix tissue of BALB/c mice was injured with angled needle under anesthesia. Mice were then inoculated with 50 *μ*L of* C. trachomatis* N or the mixture of* E. coli* and* S. aureus* (10^9^ CFU/mL). The mice were infected once a day and repeated for three days. 24 h after the last infection, the FVG was given into the mouse vagina using pipette (the volume of FVG was carefully controlled) at the dose of 2.2 g/kg, 1.1 g/kg, and 0.55 g/kg for 12 days. Policresulen suppositories (PS), as a positive control, was mixed with gel base and given at a dose of 16.5 mg/kg for 12 days. The control group and the model group were given an equal volume of gel base. On the 4^th^, 8^th^, and 12^th^ day of treatment, the cervical mucus was collected with the swab. On day 15, mice were anesthetized, blood samples were taken from aorta ventralis and the serum was separated. Mice were then killed by cervical dislocation under anesthesia and the cervix and vagina were removed. The schematic of the experimental time was shown in Figure 1.

### 2.4. ELISA Analysis of* C. trachomatis* N, IL-2, IL-17, and MCP-1

The swabs with cervical mucus were dispersed with an equal volume of PBS, the level of* C. trachomatis* N in PBS was analyzed with enzyme-linked immunosorbent assay (ELISA) kit (Meilian, Shanghai, China) according to the manufacturer's instruction (add 50 *μ*L of sample and 50 *μ*L of detection antibody to each well and incubate at 37°C for 1 hour; wash the wells, add 100 *μ*L of HRP conjugate to each well, and incubate at 37°C for 0.5 hour; wash the wells, add 50 *μ*L of chromogenic substrates to each well, and incubate at 37°C for 0.5 hour; add 50 *μ*L of stop solution to each well; read the absorbance of each well at 450 nm). The levels of IL-2, IL-17, and MCP-1in serum were determined by ELISA kit (Meilian, Shanghai, China) according to the manufacturer's instructions as described above.

### 2.5. RT-PCR Analysis of* C. trachomatis* N

Total RNA was extracted from the cervix using TRIzol (Invitrogen, California, USA); the expression of target genes was analyzed using One Step SYBR Prime Script RT-PCR Kit II (TaKaRa, Beijing, China) with Piko Real 96 (Thermo Fisher Scientific, Massachusetts, USA). The primers were used as follows: 16S rRNA forward primer, 5'-ACC CGT TGG ATT TGA GCG TA-3'; 16S rRNA reverse primer, 5'-GTT GAG CCC CGA GAT TTG AC-3'; GAPDH forward primer, 5'-GCT GAG TAT GTC GTG GAG T-3'; GAPDH reverse primer, 5'-GTT CAC ACC CAT CAC AAA C-3'. The relative expression of* C. trachomatis* N gene and mouse gene was calculated according to the 2^-ΔΔCt^ method.

### 2.6. Histological Examination

The tissues of mice were fixed with 10% formaldehyde, then embedded in paraffin and cut into slices for hematoxylin and eosin staining. The sections were visually evaluated by DMLB (Leica Camera AG, Wetzlar, Germany); two sections were prepared from each sample. The following criteria were applied for grading the pathological changes.

“-” Cervix and vagina epithelium show no hyperplasia and no inflammation and tissues are normal.

“+” Cervix and vagina epithelium have mild hyperplasia; connective tissue has mild segmental inflammation.

“++” Cervix and vagina epithelium have hyperplasia; connective tissue was infiltrated by inflammatory cells.

“+++” Cervix and vagina epithelium have significant hyperplasia and were infiltration of inflammatory cells; connective tissue was surrounded by diffuse inflammation and vascular congestion.

### 2.7. Host Cell Infection and Inclusion Stain

HeLa cells were seeded in 6-well plate with the density of 2×10^5^ cells/well and cultured for 48 h. Then the cells were incubated with* C. trachomatis* N EBs contained medium. The plate was centrifuged at 32°C for 1h and continually cultured at 37°C in the presence of 5% CO_2_ for 2h. The medium of infected cells was replaced with FE (500, 250, and 125 *μ*g/mL) contained medium and incubated for another 48 h. After then, the cells were washed with PBS, fixed with methanol and stained with Giemsa. The inclusions in HeLa cells were captured by IX71 (Olympus, Tokyo, Japan) and enumerated.

### 2.8. Statistical Analysis

Statistical analyses were performed using GraphPad Prism v.6 (GraphPad Software, California, USA). All the data were normally distributed (Kolmogorov-Smirnov test). Inclusion number and histological scores were analyzed with the Mann-Whitney* U* test; others were analyzed with the unpaired* t*-test. A value of* p* < 0.05 was considered to be significant.

## 3. Results

### 3.1. FVG Inhibits the Level of* C. trachomatis* N in Cervical Mucus

EBs of* C. trachomatis* germinate and form the RBs after invading the host cells. The RBs begin to multiply after 7-21 days. Enzyme immunoassay is a commonly used nonculture method to diagnose the chlamydial infection [9]. We analyzed the level of* C. trachomatis* N in cervical mucus to evaluate the severity of mice infection.

**Figure 2:**
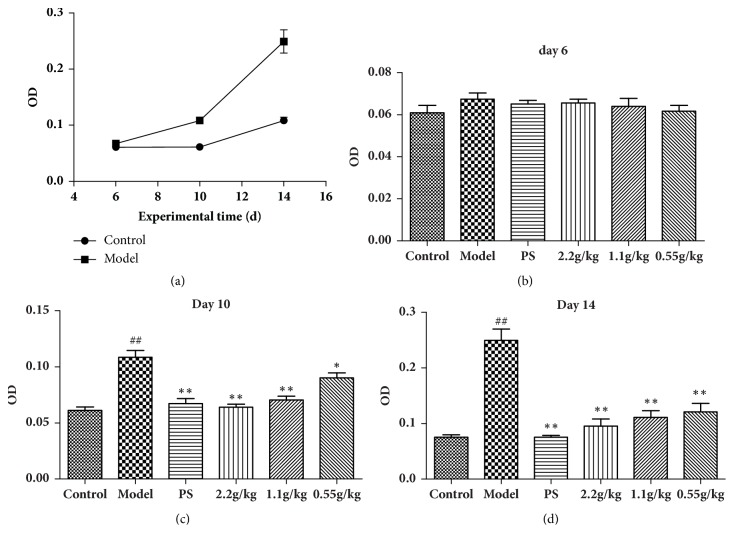
The FVG inhibit the level of* Chlamydia trachomatis* N in cervical mucus. (a) The level change of* C. trachomatis* N in the control group and the model group, (b-d) the effects of FVG on the level of* C. trachomatis* N on day 6 (b), day 10 (c), and day 14 (d). The results are mean±SEM,* n*=10, statistically significant ^##^*p*<0.01: compared with control; ^*∗∗*^*p*< 0.01 and ^*∗*^*p* < 0.05: compared with model, determined by the unpaired* t*-test.

At the early stage of* C. trachomatis* N infection, no significant change of the bacterial load was observed. 10 days after the first infection, the increase of* C. trachomatis* N in mice could be tested. On the 14^th^ day, the level of the bacterial load was significantly higher than the control group (Figure 2(a)). With the successive treatment of FVG (2.2, 1.1, 0.55 g/kg), the level of* C. trachomatis* N in mice cervical mucus could be strongly inhibited in dose-dependent manners (Figures 2(c) and 2(d)).

### 3.2. FVG Inhibits the Load of* C. trachomatis* N in Cervix

**Figure 3:**
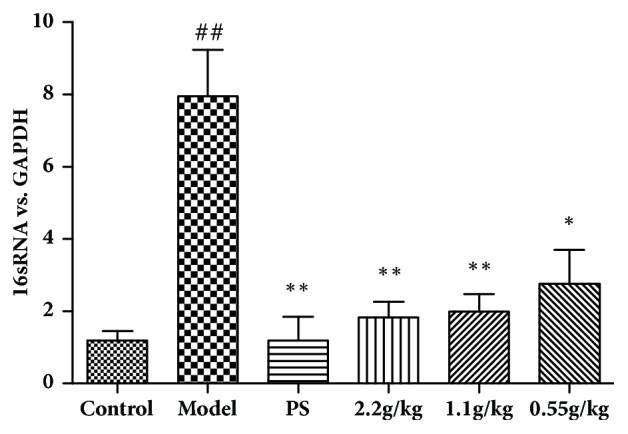
The FVG inhibit the load of* Chlamydia trachomatis* N in cervix. Relative level of* C. trachomatis* N in cervix tissues was assessed by RT-PCR. The results are mean±SEM,* n*=5, statistically significant ^##^*p*<0.01: compared with control; ^*∗∗*^*p*< 0.01 and ^*∗*^*p*< 0.05: compared with model, determined by the unpaired* t*-test.

In the diagnosis of chlamydial infection, the nucleic acid amplification technique including polymerase chain reaction (PCR) is more sensitive and specific than the enzyme immunoassay [13]. Hence, we further tested the load of* C. trachomatis* N in mice cervix with the RT-PCR method. On day 15, the expression of 16S rRNA of* C. trachomatis* N in mice was significantly higher than the control group (Figure 3). Treatment of FVG (2.2, 1.1 g/kg) for 12 days could strongly inhibit the load of* C. trachomatis* N in cervix (Figure 3).

### 3.3. FVG Diminishes the* C. trachomatis* N-Induced Cervicitis and Vaginitis

The infection of* C. trachomatis* in mice is an appropriate model for studying genital tract infections. The infection fails to induce severe upper tract genital pathology [14]. The pathology of cervix and vagina was analyzed to characterize the severity of* C. trachomatis* N infection.

**Figure 4:**
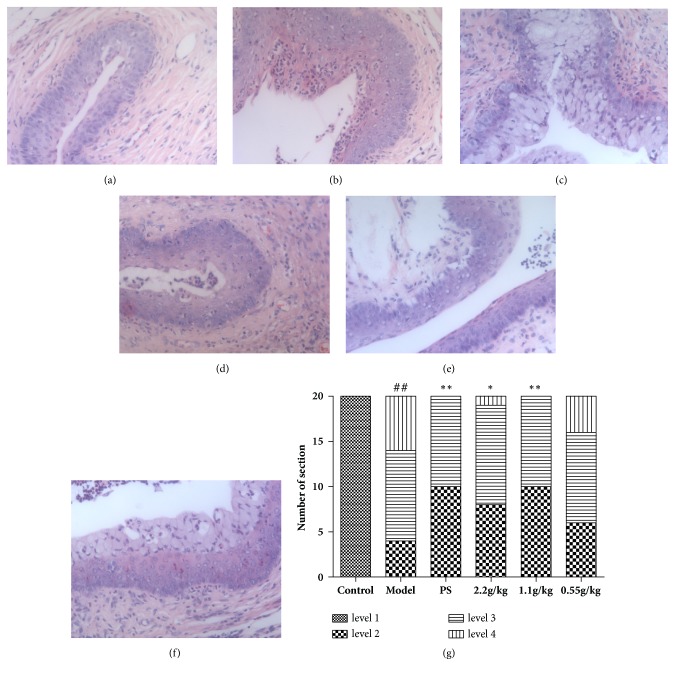
The FVG diminishes the* Chlamydia trachomatis* N-induced cervicitis. (a) Control group, (b) model group, (c) PS group, (d) FVG (2.2 g/kg) group, (e) FVG (1.1 g/kg) group, (f) FVG (0.55 g/kg) group, and (g) statistical analysis of histological examination.* n*=20, statistically significant ^##^*p*<0.01: compared with control; ^*∗∗*^*p*< 0.01 and ^*∗*^*p*< 0.05: compared with model, determined by the Mann-Whitney* U* test.

As is shown in Figure 4, in the control group, the epithelial cells and connective tissue of cervix were normal, no hyperplasia or inflammation was observed. In the model group, cervix epithelial layers have hyperplasia and the inflammatory cells were infiltrated into the epithelium and connective tissue. After the treatment of FVG (1.1 g/kg), the pathological injury of cervix could be significantly reduced (Figure 4).

**Figure 5:**
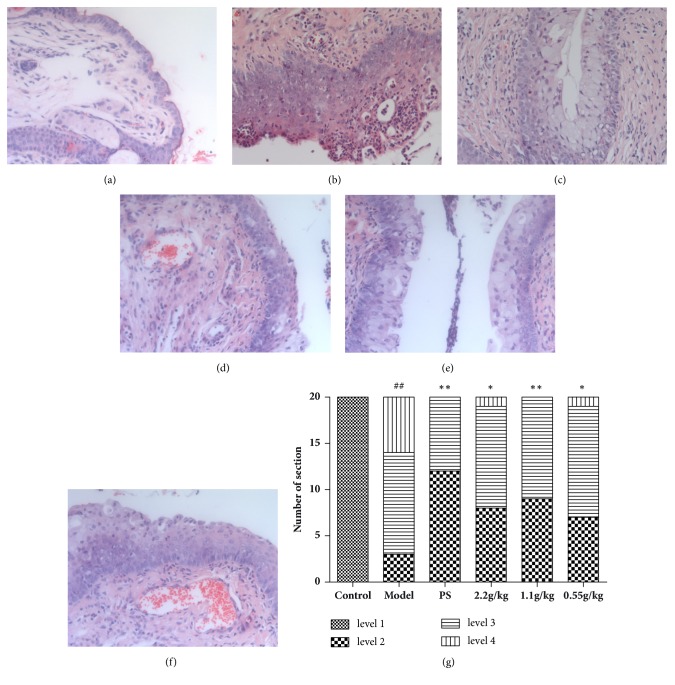
The FVG diminishes the* Chlamydia trachomatis* N-induced vaginitis. (a) Control group, (b) model group, (c) PS group, (d) FVG (2.2 g/kg) group, (e) FVG (1.1 g/kg) group, (f) FVG (0.55 g/kg) group, and (g) statistical analysis of histological examination.* n*=20, statistically significant ^##^*p*<0.01: compared with control; ^*∗∗*^*p*< 0.01 and ^*∗*^*p*< 0.05: compared with model, determined by the Mann-Whitney* U* test.

As is shown in Figure 5, in the control group, the vaginal epithelium was normal, no hyperplasia or inflammation was observed. In the model group, vaginal epithelium layers have hyperplasia and the inflammatory cells were infiltrated into the epithelium tissue. After the treatment of FVG (2.2, 1.1 g/kg), the injury of vagina could be significantly reduced (Figure 5).

### 3.4. FVG Reduces the Serum Cytokine Responses of* C. trachomatis* N Infection

**Figure 6:**
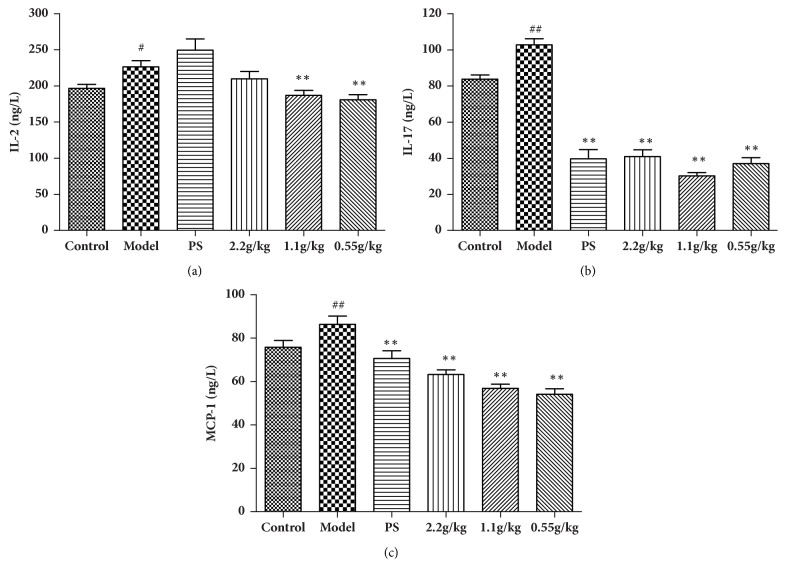
The FVG reduces the serum cytokine responses of* Chlamydia trachomatis* N infection. The levels of IL-2 (a), IL-17 (b), and MCP-1 (c) were measured by ELISA assay. The results are mean±SEM,* n*=10, statistically significant ^##^*p*<0.01: compared with control; ^*∗∗*^*p*< 0.01 and ^*∗*^*p*< 0.05: compared with model, determined by the unpaired* t*-test.

The cytokines can be released by the epithelial cells and immune cells after the* C. trachomatis* infection, most of which come from Th1 cells. These cytokines contribute to the immune responses but induce the pathological damage [15]. The levels of cytokine in serum were tested to support the histological examination results. On the 15^th^ day, the levels of IL-2, IL-17, and MCP-1 were significantly upregulated (Figure 6). Three doses of FVG could effectively inhibit the release of IL-17 and MCP-1. The production of IL-2 could be downregulated by FVG at low doses (1.1 and 0.55 g/kg).

### 3.5. FVG Reduces the* C. trachomatis* N-Induced Inclusion Count* In Vitro*

**Figure 7:**
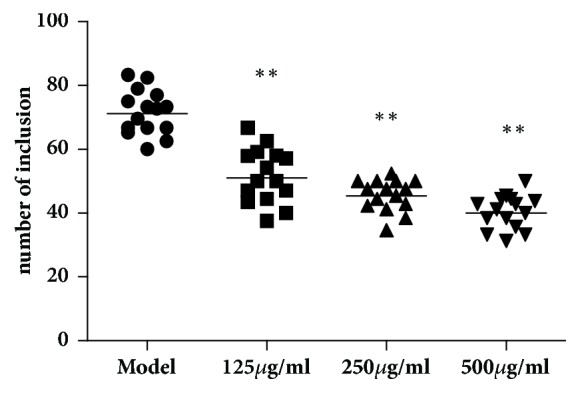
The FVG reduces the* Chlamydia trachomatis* N-induced inclusion count* in vitro*. The number of inclusion was enumerated after being stained by Giemsa.* n*=15, statistically significant ^*∗∗*^*p*< 0.01: compared with model, determined by the Mann-Whitney* U* test.

The proliferation of* C. trachomatis* occurs in the host cells with the formation of RBs. Inclusion can be formed approximately 12 h after infection, which contains numbers of RBs [15]. The* in vitro* inhibition of FVG on the infection of* C. trachomatis* N was evaluated with the number of inclusion. After HeLa cells being incubated with* C. trachomatis* N for 48h, inclusions could be observed with the Giemsa stain. After treatment with FVG (FE), the number of inclusion was significantly downregulated in a dose-dependent manner (Figure 7).

### 3.6. FVG Reduce the Pathological Injury of Cervix Induced by* E. coli* and* S. aureus*

Even though* C. trachomatis* is reported to be the most common pathogen of cervicitis, anaerobes such as* E. coli*,* S. aureus*, and* Klebsiella pneumoniae* could be isolated in the cervicitis patients [5, 16]. A rat cervicitis model infected by the mixture of* E. coli*,* S. aureus*, and* N. gonorrhea* had been established in our previous work [17]. In the present research, a mice model infected by the mixture of* E. coli* and* S. aureus* was optimized. 14 days after the first infection, the cervix was proved to be severely infected.

**Figure 8:**
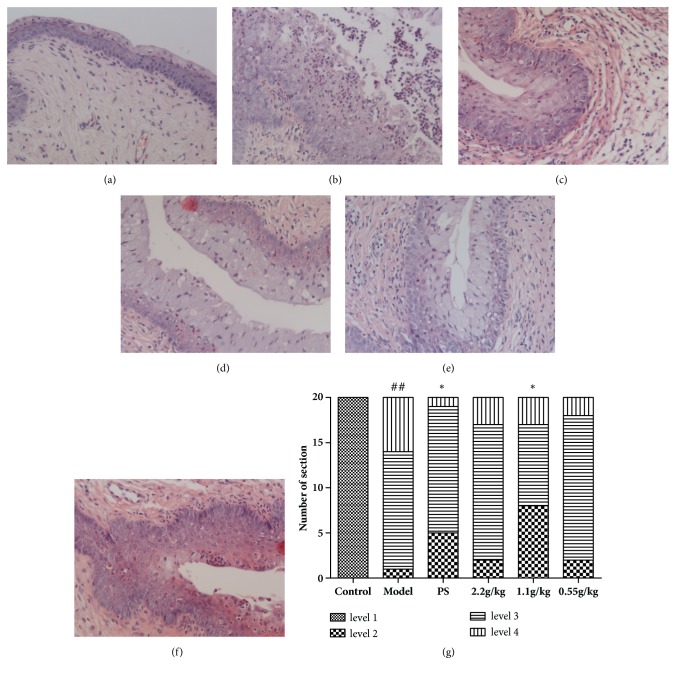
The FVG reduce the pathological injury of cervix induced by* Escherichia coli* and* Staphylococcus aureus*. (a) Control group, (b) model group, (c) PS group, (d) FVG (2.2 g/kg) group, (e) FVG (1.1 g/kg) group, (f) FVG (0.55 g/kg) group, and (g) statistical analysis of histological examination.* n*=20, statistically significant ^##^*p*<0.01: compared with control; ^*∗∗*^*p*< 0.01 and ^*∗*^*p*< 0.05: compared with model, determined by the Mann-Whitney* U* test.

In the control group, the cervical epithelium was normal and no hyperplasia or inflammation was observed. In the model group, inner layers of cervical epithelium have hyperplasia, and the inflammatory cells (neutrophil and eosinophil) were severely infiltrated into the epithelium tissue. After the treatment of FVG (2.2 and 1.1 g/kg), the inflammation of cervix could be significantly inhibited (Figure 8).

## 4. Discussion

The diagnosis of cervicitis is difficult due to the lack of the obvious symptom. In some patients, abnormal mucopurulent discharge and cervical ectopy could be observed [3]. Many mammal models of cervicitis had been established in the last decades. Macaques, rats, and mice could be used to establish the cervicitis model. Both bacteria (*C. trachomatis*) and chemical compounds (phenol, acetic acid) could be used to induce the cervicitis [17–20]. In this research, two mouse models were optimized. After infection with* C. trachomatis *N, the bacterial load of* C. trachomatis* N in cervical mucus was continuously raised over time. The* C. trachomatis *N infection could lead to the upregulation of cytokines in serum and the inflammation in the tissues of vagina and cervix. After infection with the mixture of* E. coli* and* S. aureus*, the injury of cervix could be detected in pathologic diagnosis.

Clinically, antibiotics were the first choice to treat cervicitis according to different pathogens. But the emergence of antibiotic-resistant such as the quinolone-resistant* C. trachomatis *and* N. gonorrhea*, methicillin-resistant* S. aureus*, and vancomycin-resistant* E. coli* becomes a serious problem worldwide due to the abuse of antibiotics [4, 5, 9]. The TCM may contribute alternative strategies for the treatment of cervicitis. The effects of FVG on cervicitis were tested in this research.

FVG is a vaginal gel, the gel base of which is the mixture of carbopol 941 and xanthan gum [12]. In recent years, many commercial vaginal gel preparations of Chinese herbal formula have been used to treat the cervicitis [21]. The vaginal drug delivery is a traditional mucosal drug delivery route, which can be used for the treatment of both local and systemic diseases [22]. Traditional preparations, such as suppositories, gels, tablets, vaginal films, irrigations, and pessaries can be used as vaginal formulations [22, 23]. As one of the most widely used drug delivery systems, vaginal gels have been used for the preparation of microbicides, contraceptives, labor inducers, and sex hormones. Comparing with other drug delivery systems, the vaginal gel is safer and has higher bioavailability [24].

All the six herbal medicines in FVG can be used in treating gynecological diseases in TCM [11]. According to the theory of TCM, in this formula, the Gentianae radix et rhizome is the “monarch drug” or “principal drug”, the Stemonae radix and Fraxini cortex are the “ministerial drug” or “assistant drug”, the Paeoniae radix rubra and Dictamni cortex are the “adjuvant drug”, and the Glycyrrhizae radix et rhizome is the “guiding drug”. The Gentianae radix et rhizome may contribute the most to the effects of FVG. Iridoids and total glucosides of peony are mainly isolated from Gentianae radix et rhizoma and Paeoniae radix rubra, respectively. These compounds show significant anti-inflammatory effects [25, 26]. Alkaloids and limonoids contribute to the antibacterial activity of Dictamni cortex [27]. The coumarins in Fraxini cortex and the flavones in Glycyrrhizae radix et rhizome show antibacterial and anti-inflammation effects [28, 29]. The alkaloids contribute to the insecticidal activity of Stemonae radix [30]. These bioactive compounds of these herbal medicines may support the treatment of FVG on cervicitis.

IL-17 is produced by Th17 cells characteristically. Clinically, the concentration of IL-17 in genital secretions of* C. trachomatis* infected patients is significantly higher than that of uninfected women [31]. The high level of IL-17 in the mouse cervicitis model was in agreement with the clinical result. The levels of IL-2 and MCP-1 are associated with the responses of Th1. IL-2 is secreted from Th1 cells and stimulates the production of cytokines. There is an inverse correlation between the level of MCP-1 and the Th1 responses. The inhibition of the MCP-1 can increase the production of IFN-*γ* [32, 33]. The FVG could downregulate the concentrations of IL-2, IL-17, and MCP-1. These results suggested that FVG could inhibit the cervicitis through the regulation of host immune responses.

Early in the* C. trachomatis* cycle of infection, type III secretion system enables the EBs to invade the host cells [34]. The internalization of* C. trachomatis *is followed by the development of RBs and inclusions. The inhibitor of type III secretion system could block the formation of inclusions [35]. The* in vitro* investigation demonstrated that FVG could inhibit the number of inclusion in HeLa cells.

In conclusion, we successfully established two mouse cervicitis models with the infection of* C. trachomatis *N,* S. aureus*, and* E. coli*. We proved that FVG could significantly inhibit the cervicitis. FVG could downregulate the bacterial load, mitigate the pathological injury, and reduce the number of inclusion. The effects of FVG were associated with the inhibition of pathogens and the regulation of host immune responses. This research provides evidence that FVG could be used as a novel alternative agent that alleviates the problem of antibiotic-resistant during the treatment of cervicitis.

## Data Availability

The data used to support the findings of this study are included within the article.
